# Inhibition of growth and induction of apoptosis in human breast cancer by transfection of *gef* gene

**DOI:** 10.1038/sj.bjc.6601064

**Published:** 2003-07-01

**Authors:** H Boulaiz, J Prados, C Melguizo, Á M García, J A Marchal, J L Ramos, E Carrillo, C Vélez, A Aranega

**Affiliations:** 1 Basic Cardiovascular Research Section, Department of Morphological Sciences, School of Medicine, University of Granada, E-18012 Granada, Spain; 2 Department of Health Sciences, University of Almería, E-04002 Almería, Spain; 3 Clinical Analysis Service, Virgen de las Nieves Hospital, E-18014 Granada, Spain; 4 Department of Health Sciences, University of Jaén, E-23071 Jaén, Spain; 5 Zaidín Experimental Station, CSIC, E-18008 Granada, Spain

**Keywords:** gene therapy, *gef* gene, breast cancer, apoptosis

## Abstract

The *gef* gene has cell-killing functions in *Escherichia coli*. To evaluate the feasibility of using this gene as a new strategy for cancer therapy, we transfected it in MCF-7 cells derived from breast cancer (MCF-7TG). The *gef* gene was cloned in a pMAMneo vector under the control of a mouse mammary tumour virus promoter, inducible by dexamethasone (Dex), and was transfected with liposomes. After selection and induction, expression of the *gef* gene was confirmed by reverse transcription–polymerase chain reactions (RT–PCR) and Western blot. Cell viability was determined with a haemocytometre and the sulphorodamine B colorimetric assay, and the cell cycle was studied by propidium iodide (PI) staining. Annexin V-FITC and PI assays were used to evaluate apoptosis, which was confirmed by electron microscopy. In comparison with MCF-7 parental cells and MCF-7 cells transfected with an empty vector, MCF-7TG cells induced with Dex showed a significant decrease in proliferation rate, which was associated with evidence of apoptosis. Morphological findings confirmed apoptosis and showed a typical pattern of mitochondrial dilation. Furthermore, the cell cycle was characterised by premature progression from G_1_ to S phase and G_2_ delay. Our results show that the *gef* gene was able to decrease proliferation in a breast cancer cell line, and induce apoptosis. These findings suggest that the *gef* gene is a potential candidate for tumour therapy.

There is an urgent need to develop novel systemic strategies for patients with cancer. Gene transfer to tumour cells offers new possibilities for cancer therapy based on the genetic modification of tumour cells (Marchisone *et al*, 2000). Strategies based on putative killer-suicide genes such as *Herpes simplex* virus thymidine kinase and cytosine deaminase indirectly induce cytotoxicity in mammalian cells. In both systems, the enzymes encoded by suicide genes convert a nontoxic prodrug (ganciclovir and 5-fluorocytosine, respectively) into a toxic metabolite (Shangara *et al*, 2000), which causes DNA chain termination or inhibits RNA and DNA synthesis during the S phase of the cell cycle (Greco and Dachs, 2001). However, problems with the release of toxic metabolites have led to the development of new strategies for cancer therapy based on the use of toxic genes, which do not need prodrugs to be effective in tumour cells. These promising strategies aim to act directly on the cell cycle and cell proliferation (Pietersen *et al*, 1999; Martin *et al*, 2000; Pützer *et al*, 2000). The expression of these genes is controlled by a significant number of tumour tissue-selective promoters and enhancer elements that have demonstrated their potential for use in gene therapy. However, promoters with specific actions in different cancers will need to be selected to realise the potential of this new therapeutic approach (Hart, 1996; Shillitoe *et al*, 2000).

In this context, a number of genes have been identified as members of a gene family that encodes homologous cell-killing functions in *Escherichia coli* (Gerdes *et al*, 1990). In a member of this family, named the *gef* gene (isolated from the chromosome of *E. coli*) (Poulsen *et al*, 1991), the killing function is mediated by a membrane protein of about 50 amino acids, which induces the arrest of cellular respiration; subsequently, the cells undergo morphological changes (characteristic ‘ghost’ cells) and become nonviable (Poulsen *et al*, 1992). Previous studies have demonstrated that Gef is anchored in the cytoplasmic membrane by the N-terminal part of the protein, whereas the C-terminal part is localised in the periplasm. Mutagenesis experiments with Gef showed that the periplasmic portion of the gene encodes the toxic domain, and that dimerisation is not essential for the toxic effect (Poulsen *et al*, 1991). This effect was expressed in analyses with suicide cassettes consisting of members of the *gef* gene family in combination with inducible promoters (Molin *et al*, 1993). This gene, currently being studied to determine its ability to control bacterial population death (Ronchel and Ramos, 2001), may be a new candidate for therapeutic applications.

In this study, we used a mammalian expression vector containing the *gef* gene under the control of the mouse mammary tumour virus (MMTV) promoter, inducible by dexamethasone (Dex), to evaluate the expression, *in vitro*, of this gene in tumoral MCF-7 cells derived from breast cancer. We found that expression of the *gef* gene in these tumoral cells significantly decreased growth and induced apoptosis. These results suggested the feasibility of using a *gef* gene system as a new strategy for cancer therapy.

## MATERIALS AND METHODS

### Construction of *gef* expression vector

The *gef* gene (kindly provided by the Zaidín Experimental Station, CSIC, Granada, Spain) was subcloned into the mammalian expression vector pMAMneo (Clontech, Palo Alto, CA, USA), which allows Dex-inducible expression of cloned inserts. The full-length *gef* insert, flanked at 3′ and 5′ by a *Xho*I and an *Nhe*I restriction site, respectively, was amplified by polymerase chain reaction (PCR) with routine DNA manipulation (Sambrook *et al*, 1989). The PCR product was double-digested with *Nhe*I and *Xho*I and inserted into pMAMneo, and this construction was used to transform *E. coli*. The cells were plated onto Luria–Bertani broth plates containing ampicillin. A single insert-positive clone (with pMAMneo-*gef*) was selected for transfection into MCF-7 cells after analysis by restriction-enzyme digestions and PCR to establish reading frame orientation and sequence fidelity.

### Cell culture and stable transfection

The human breast cancer MCF-7 cell line was kindly provided by Dr N Olea of the Sánchez Mora Tumoral Biology Institute, University Hospital of Granada. MCF-7 cells were grown at 37°C in an atmosphere containing 5% CO_2_, with Dubelcco's modified Eagle's medium (DMEM) (Gibco, Grand Island, NY, USA) supplemented with 10% heat-inactivated foetal bovine serum (FBS) (Gibco), 2% L-glutamine, 2.7% sodium bicarbonate, 1% Hepes buffer, 40 mg l^−1^ gentamicin and 500 mg ampicillin^−1^. To isolate stably transfected cells, 2 days before transfection confluent MCF-7 cells were split 1 : 5 into six-well plates, yielding approximately 70% confluence on the day of transfection. The cells were washed with culture medium, then transfected using 6 *μ*l FuGENE 6 reagent (Roche Diagnostic, Barcelona, Spain) with 1.8 *μ*g per well of pMAMneo empty vector or pMAMneo-*gef* in 2 ml of culture medium. After 48 h, the medium was replaced with selective medium: DMEM, 10% FBS, 500 *μ*g ml^−1^ geneticin G418 (Sigma, St Louis, MO, USA), and resistant clones were selected. Single clones of stably transfected cells, isolated by limiting dilution in 96-well plates, were transferred to individual flasks and cultured in medium containing 300 *μ*g ml^−1^ G418. For the present study, a clone named MCF-7TG (MCF-7 cells transfected with pMAMneo-*gef* gene) was used. Integrity of the transfected vector sequence in the MCF-7 cells was analysed by PCR. Genomic DNA was extracted from MCF-7 parental and MCF-7TG cells with the Magic™ Megapreps DNA Purification System (Promega, Madison, WI, USA) according to the manufacturer's instructions. Total DNA (250 ng) was diluted to 50 *μ*l in a mixture with a final concentration of 250 *μ*M dNTP, 100 ng of each primer, 5 *μ*l PCR buffer and 2.5 U *Taq* polymerase (Roche Diagnostic). The primers used were Gef 3: 5′GAAGCAGCATAAGGCGATG3′ and Gef 4: 5′CTCGG ATTCGTAAGCCGTG3′. Polymerase chain reaction (94°C for 5 min, 61°C for 1 min and 72°C for 1 min) was carried out for 32 cycles with a final elongation step of 72°C for 10 min. For analysis, 10 *μ*l of the reaction product was run on a 2% agarose gel, visualised by ethidium bromide staining and photographed under ultraviolet light.

### Reverse transcription (RT)–PCR and Western blot analysis

For RT–PCR analysis, total RNA was extracted from MCF-7 and MCF-7TG cells after 24 h of induction with Dex with an RNA extraction kit (Promega) according to the manufacturer's instructions. RNA (1 *μ*g) from each cell line was then reverse-transcribed into cDNA using a first-strand cDNA synthesis kit (Promega). PCR of the RT was done and analysed under the same condition as detailed above, using 5 *μ*l of the RT products. The integrity of RNA was assessed by amplification of *β*-actin mRNA using 5′-primer nt 1854–1873 and 3′-primer nt 2151–2170 (Prados *et al*, 1998). For Western blot analysis, we used a polyclonal antibody against the Gef protein that was produced at the Technical Service of the University of Seville (Seville, Spain) by immunising rabbits with a 25 amino-acid synthetic peptide (carboxy terminus of the protein) predicted from the DNA sequence of the *gef* gene (Poulsen *et al*, 1991). Total proteins were obtained from MCF-7 and MCF-7TG cells after induction for 24 h with Dex. The cells were harvested and lysed in sample buffer consisting of 62.5 mM Tris HCl (pH 6.8), 10% glycerol, 2% SDS, 5% 2-mercaptoethanol, 0.5% bromophenol blue and 100 mM dithiothreitol. Proteins were separated on 15% SDS–polyacrylamide gels and electroblotted onto Immobilon-P-membranes (Millipore, Bedford, MA, USA). Blocking, washing, and incubations of the blotted nitrocellulose membranes were carried out as described (Prados *et al*, 1995). The polyclonal antibody against the Gef protein was diluted 1 : 10, and positivity was detected with an anti-rabbit IgG peroxidase conjugate (Sigma).

### Cell proliferation assays

MCF-7 and MCF-7TG cells (25 × 10^3^) were plated into six-well plates under the culture conditions detailed above. After 24 h, cells were fed with fresh medium and treated with 1 *μ*M Dex (Sigma) to induce the promoter activity of MMTV. In the control groups, cells were not treated with dexamethasone. To verify that Dex had no effect on cell growth, MCF-7 parental cells were also treated with Dex. The cultures were fed with fresh medium with or without Dex every alternate day up to the end of the experiment; each treatment and time point was run in four plates. After 1, 2, 4, 6, 10 and 15 days of treatment, cells were trypsinised, collected, and counted with a haemocytometre. Trypan blue dye exclusion was used to determine cell viability. The same experiment was performed using the sulphorodamine-B (SRB) colorimetric assay as described previously (Villalobos *et al*, 1995), with a Titertek Multiscan apparatus (Flow, Irvine, CA, USA) at 492 nm. We evaluated linearity of the colorimetric results with cell number for each MCF-7 cell stock before each cell growth experiment. MCF-7 cells transfected with pMAMneo empty vector were used in the proliferation assay as a control.

### Apoptosis detection by staining with annexin V-FITC and propidium iodide (PI)

The Annexin V-FITC Apoptosis Detection Kit I (Pharmingen, San Diego, CA, USA) was used to detect apoptosis by flow cytometry. MCF-7TG cells (1 × 10^6^ cells) were plated onto 75-cm^2^ flasks and cultured overnight, followed by incubation with Dex for 48 and 96 h. MCF-7 parental cells and MCF-7TG cells were harvested by phosphate-buffered saline-ethylenediamine-tetraacetic acid (PBS-EDTA), washed twice in cold PBS (1.4 M NaCl, 27 mM KCl, 100 mM KH_2_PO_4_/K_2_HPO_4_, pH 7.2), and pelleted by centrifugation at 500 **g** for 10 min. They were then resuspended at 10^6^ cells 100 *μ*l^−1^ in a binding buffer (Hepes buffer, 10 mM, pH 7.4, 150 mM NaCl, 5 mM KCl, 1 mM MgCl_2_, 1.8 mM CaCl_2_), stained with annexin V incubation reagent (1 *μ*l annexin V-FITC (25 *μ*g ml^−1^), 10 *μ*l binding buffer, 10 *μ*l PI (50 *μ*g ml^−1^, 79 *μ*l H_2_O) and incubated in the dark for 15 min at room temperature. Then, 500 *μ*l binding buffer was added and the cells (10 000 cells per sample) were immediately processed with a FACScan flow cytometre (Becton Dickinson, San Jose, CA, USA).

### Ultrastructural analysis

Adherent and nonadherent MCF-7 parental cells and MCF-7TG cells treated with Dex during 48 and 96 h were fixed with 2.5% glutaraldehyde in 0.1 M sodium cacodylate buffer (pH 7.4) for 1 h at room temperature. The pellet and monolayer were postfixed with 1% osmium tetroxide in 0.1 cacodylate buffer for 1 h at room temperature, and dehydrated in ethanol. Cells were detached from the culture vessel by rapid treatment with propylene oxide and embedded in Epon 812. After polymerisation, the plastic was removed and ultrathin sections were cut parallel and perpendicular to the surface of the flask. The sections were contrasted with uranyl acetate–lead citrate and examined in a Hitachi H7000 transmission electron microscope.

### Cell cycle analysis

Cells in monolayer culture were harvested, washed twice with sample buffer (100 mg glucose; 100 ml PBS without Ca^2+^ or Mg^2+^) and fixed in 70% (vol vol^−1^) cold ethanol for up to 1 week. Cells were pelleted, washed once with sample buffer and resuspended in PI solution (50 *μ*g ml^−1^ PI, 0.5 mg ml^−1^ RNase in sample buffer, pH 7.4) for 30 min in the dark. Fluorescence-activated cell sorter analysis was performed 48 and 96 h after induction. The data from 10 000 cells per sample were collected and analysed using the Cellfit program on a FACScan flow cytometre (Becton Dickinson, San Jose, CA, USA).

### Statistical analysis

All statistical analyses were carried out with the SPSS, release 7.5 (SPSS, Chicago, IL, USA). The results were compared with Student's *t*-test. All data are expressed as the mean±s.d. The differences were considered statistically significant at a *P*-value of <0.05.

## RESULTS

### Gef protein expression in MCF-7 transfected cells

**Figure 1:**
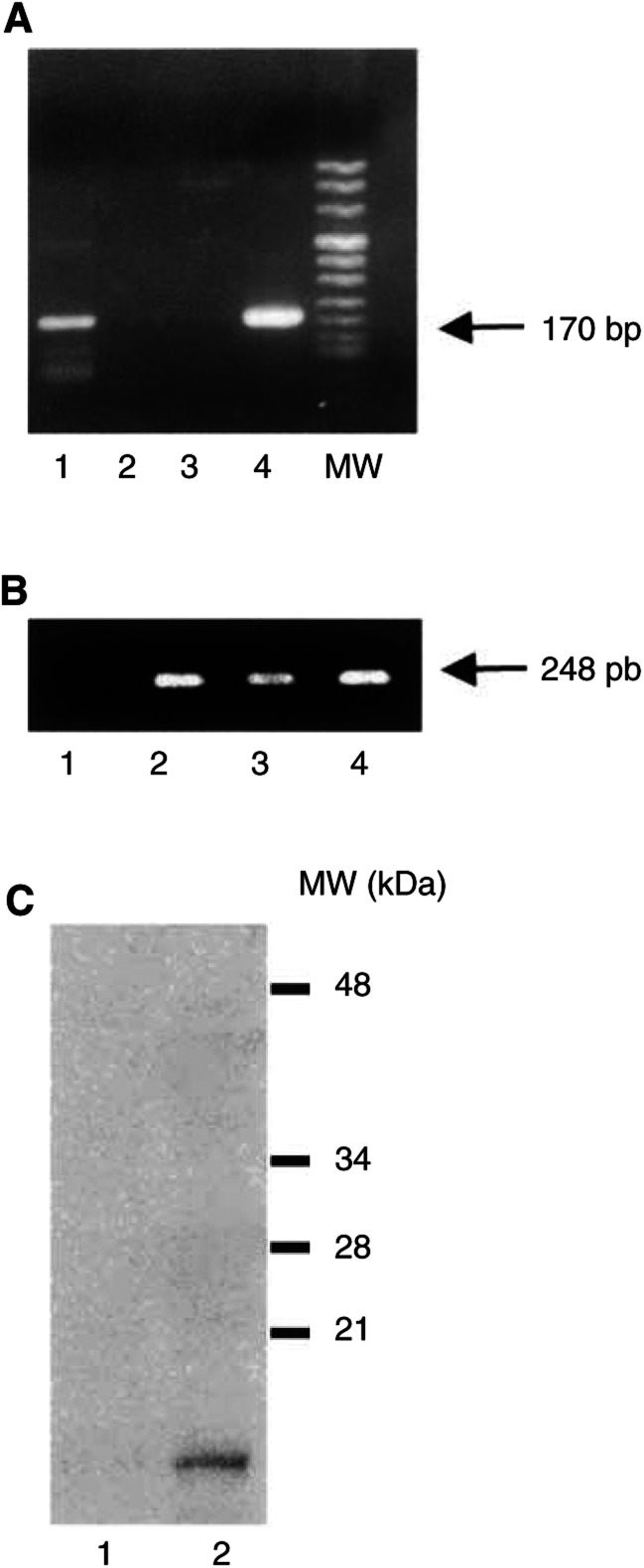
Determination of *gef* gene expression by RT–PCR and immunoblot. Total RNA isolated from MCF-7 parental and MCF-7TG cells was transcribed to cDNA using reverse transcriptase. Polymerase chain reaction amplification of cDNA was performed as described in Materials and Methods. The amplified PCR product of *gef* mRNA (a) and *β*-actin mRNA (b) were separated by 2% agarose gel electrophoresis and visualised with ethidium bromide. (**A**) Lane 1, MCF-7; lane 2, MCF- 7TG; lane 3, pMAMneo-*gef* (positive control). (**B**) Lane 1, MCF-7; lane 2, MCF-7TG; lane 3, pMAMneo-*gef* (negative control). (**C**) Western blot analyses of total proteins prepared from MCF-7 parental and MCF-7TG cells were performed with a polyclonal antibody against the Gef protein. Lane 1, MCF-7; lane 2, MCF-7TG. MW, molecular weight VIII.

To analyse the effect of the *gef* gene in tumoral cells, MCF-7 cells were transfected with the pMAMneo inducible vector containing the *gef* sequence. The presence and integrity of DNA from the *gef* gene in MCF-7 transfected cells (MCF-7TG) was assessed by PCR with genomic DNA (data not shown). The gene was expressed in cultures of MCF-7TG cells that contained Dex, but not in those without Dex. The presence of mRNA from the *gef* gene in MCF-7TG cells was verified by RT-PCR (Figure 1

). Western blot analyses were performed to determine the presence of the Gef protein in MCF-7TG cells. The results of immunoblotting with the polyclonal antibody against the Gef protein were consistent with the RT–PCR results (Figure 1).

### Modulation of growth pattern in MCF-7 transfected cells

**Figure 2:**
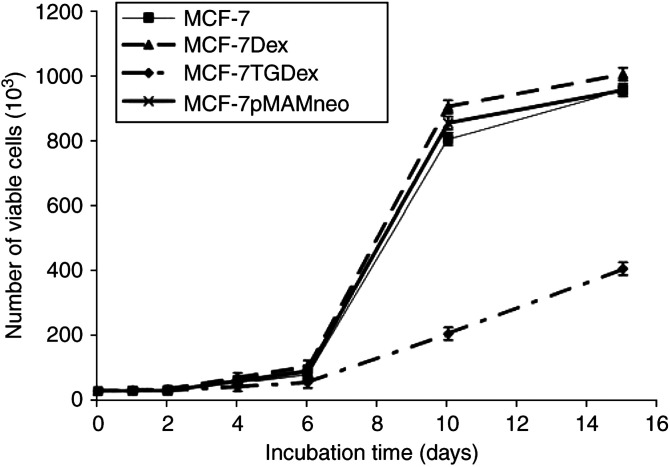
Determination of the effects of *gef* gene expression on cell proliferation. MCF-7 parental cells, MCF-7 cells induced with Dex (MCF-7Dex), MCF-7TG cells induced with Dex (MCF-7TGDex) and MCF-7 cells transfected with empty vector (MCF7-pMAMneo) were seeded at 25 × 10^3^ cells per dish and cultured for 15 days to determine the growth rate. Values represents means±s.d. of quadruplicate cultures.

We found no significant differences in growth patterns between MCF-7 parental cells with or without Dex treatment and MCF-7 cells transfected with pMAMneo empty vector. In MCF-7 cells transfected with *gef* and treated with Dex, the decrease in proliferation rate in comparison with parental MCF-7 was first observed on day 4 of induction, became significant by day 6, and was obvious by day 10 of culture (Figure 2

). After 48 h of induction, growth was not inhibited in MCF-7TG cells compared to MCF-7 parental cells or those transfected with empty vector. At 6 days, however, MCF-7TG cells incubated with 1 *μ*M Dex showed a 34.4% decrease in growth (*P*<0.001) with respect to control cultures. After 10 and 15 days of incubation with Dex, growth was inhibited by 75 and 67.9%, respectively (*P*<0.001).

### Apoptosis in MCF-7 transfected cells

**Figure 3:**
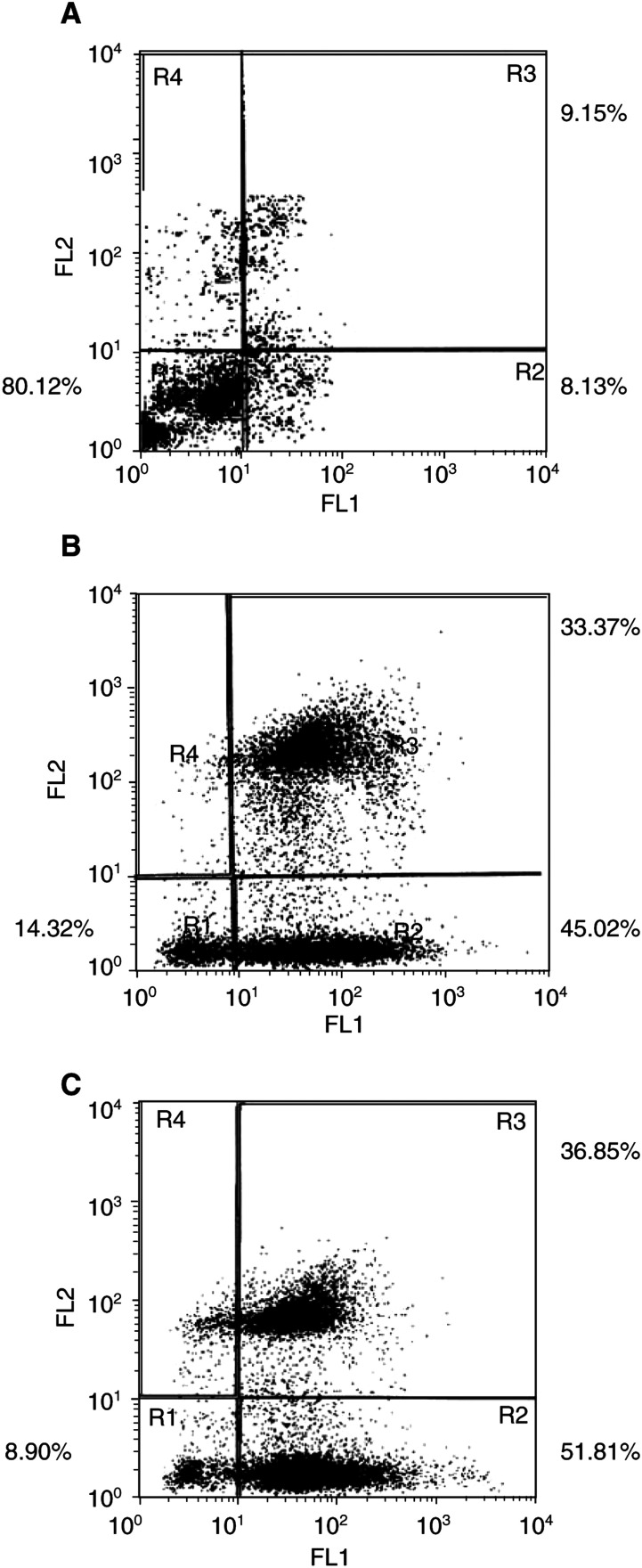
Quantification of *gef*-induced apoptosis in MCF-7TG cells by fluorescence-activated cell sorting analysis. Cells were stained with annexin V and PI to evaluate apoptotic cell death, as described in Materials and Methods. (**A**) MCF-7 cells. (**B**) MCF-7TG cells after 48 h of induction with Dex. (**C**) MCF-7TG cells after 96 h of induction with Dex. The data shown are representative of three separate experiments.

Simultaneous staining with annexin V-FITC and IP nonvital dye made it possible to distinguish between intact cells (stained negative for both annexin V-FITC and PI), early apoptosis (stained positive for annexin V-FITC and negative for PI), and late apoptosis or cell death (stained positive for both and annexin V-V-FITC and PI). In our MCF-7 control culture, 80.12% of the cells were viable, 8.13% were in early apoptosis, and 9.15% were in the late or final stages of apoptosis (*P*<0.001) (Figure 3a

). Initial analysis of MCF-7 cells induced with Dex during 12 and 24 h showed no significant modification in relation to the control culture (data not shown). However, in MCF-7TG cells treated with Dex during 48 h, 14.32% of the cells were viable, 45.02% were in early apoptosis and 33.37% were in the late or final stages of apoptosis (Figure 3b). In MCF-7TG cells treated with Dex for 96 h, the corresponding figures were 8.90, 51.81 and 36.85% (*P*<0.001) (Figure 3c).

### Morphological analysis of MCF-7 transfected cells

**Figure 4:**
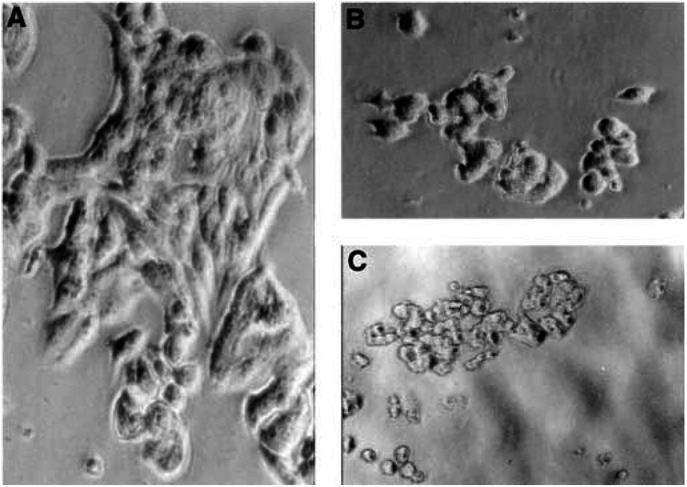
Phase contrast micrographs of parental and transfected MCF-7 cells. Parental MCF-7 cells, grew in clumps, were typically polygonal in outline (× 40) (**A**) and adhered strongly to the culture flask, whereas MCF-7TG cells treated with Dex for 48 h (× 40) (**B**) to induce *gef* gene expression were rounded and easily detached from the culture flask. These changes were more marked in MCF-7TG cells after 96 h of Dex induction (× 20) (**C**).

**Figure 5:**
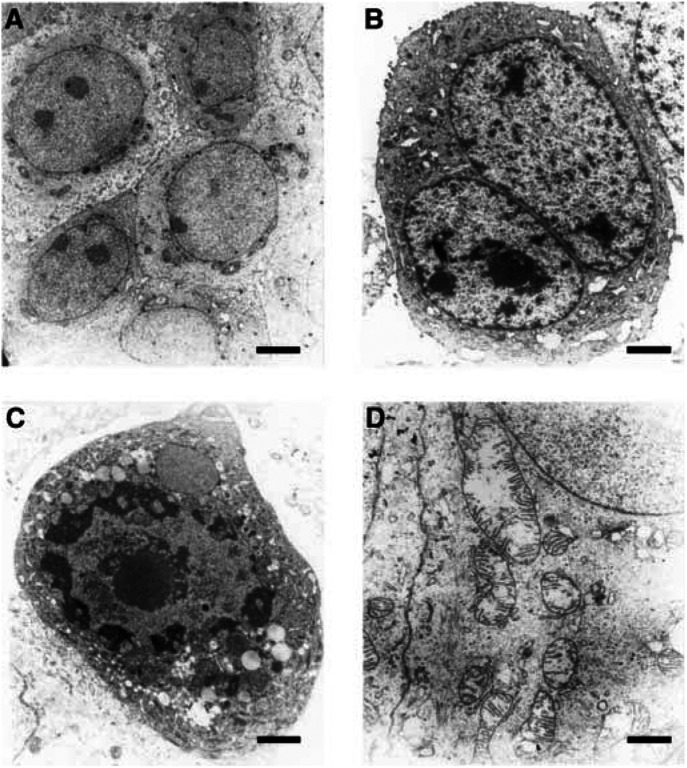
Ultrastructural morphology of MCF-7 and MCF-7TG cells treated with Dex to induce *gef* gene expression. (**A**) Typical morphology of MCF-7 parental cells demonstrated light cytoplasmic complexion (× 2500). (**B**) MCF-7TG cell with condensed granular-appearing chromatin (× 4000). (**C**) Dying MCF-7TG cell with a convoluted nuclear membrane. The masses of compact chromatin are highly electron dense and homogeneous in texture. Note mininuclei with a different chromatin pattern (× 6300). (**D**) Detail of cytoplasm of an MCF-7TG with mitochondrial dilation (× 8000). Scale bars: (**A**) 2.4 *μ*m; (**B**) 1.5 *μ*m; (**C**) 0.9 *μ*m; (**D**) 0.75 *μ*m.

Light microscopic observations showed modifications in the morphology of MCF-7TG cells after induction with Dex, characterised by rounding and loss of adherence to the flask (Figure 4

). MCF-7 cells transfected with empty vector showed no morphological changes in comparison with the parental cell line. Moreover, Dex had no effect on the morphology of MCF-7 parental cells (data not shown). However, the death of MCF-7TG cells by apoptosis, which appeared after 48 h of Dex treatment, was clearly confirmed in ultrastructural images. In the cell nucleus, the earliest recognisable cytological changes were compaction and segregation of the chromatin at the nuclear periphery. Initially, the fine structure of this chromatin was similar to that of condensed chromatin clumps in healthy cells, with the granular appearance of cross-sectioned chromatin fibres being clearly visible (Figure 5b

). At more advanced stages of apoptosis, progression of chromatin compaction was associated with redistribution of chromatin fibres, resulting in electron-dense domains of degraded homogeneous-appearing chromatin. In some instances compaction and degradation of chromatin occurred in association with convolution of the nuclear profile (Figure 5c). Another important feature within the nucleus of apoptotic cells was the reorganization of ribonucleoprotein-containing structures, including the nucleolus. Compaction and marginalisation of the chromatin produced clear nucleoplasmic areas in the nucleus (Figure 5c). In the cytoplasm, we observed dilated mitochondria (Figure 5d).

### Modulation of cell cycle in MCF-7 transfected cells

**Figure 6:**
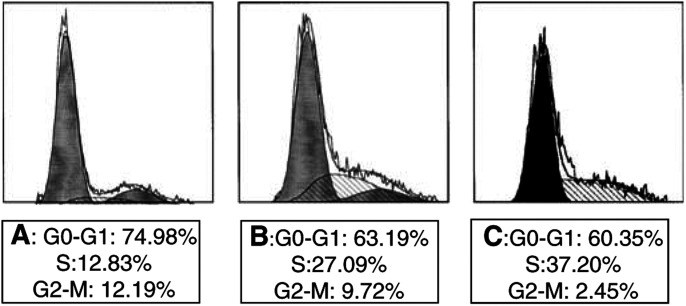
FACScan analysis of the cell cycle. MCF-7 and MCF-7TG cells treated with Dex were harvested and analysed to distinguish populations of cells in different phases of the cycle, as described in Materials and Methods. (**A**) Parental MCF-7 cells. (**B**) Transfected MCF-7TG cells after 48 h of Dex induction. (**C**) Transfected MCF-7TG cells after 96 h of Dex induction.

Parental cell cultures contained 74.98% G_1_/G_0_ cells, 12.83% S-phase cells and 12.19% G_2_/M-phase cells. In contrast, after 48 h of Dex induction, MCF-7TG cells showed a gradual disappearance of G_1_-phase cells and a corresponding accumulation of S-phase cells. After 96 h of induction, the increase in the S-phase population (37.20%) and decrease in the number of G_1_ phase cells (60.53% G_1_/G_0_ cells) and G_2_/M-phase cells (2.45%) were even more marked (Figure 6

).

## DISCUSSION

We demonstrate for the first time that transfection of the *gef* gene makes it possible to decrease tumour cell growth in breast cancer cell line MCF-7. Our system is independent of the administration of a prodrug, which is required in systems such as *Herpes simplex* virus thymidine kinase and cytosine deaminase (Greco and Dachs, 2001), and which may present problems related to general toxicity, drug release and bioviability.

The use of genes that damage tumour cells and do not require a prodrug is one of the aims of new gene therapy strategies. Recently, some procaryotic and viral genes have been shown to be able to induce tumour cell death (Martin *et al*, 2000; Zhuang *et al*, 1995; Paulus *et al*, 1997). In this connection, the *gef* gene shows a killing function in procaryotic cells, which may be modulated by the action of different promoters (Molin *et al*, 1993). The product of the *gef* gene is a cytoplasmatic membrane protein whose 19 N-terminal hydrophobic amino acids constitute a membrane-spanning segment, probably in the form of an alpha-helix structure that anchors the protein to the membrane. The C-terminal part, localised in the periplasm in dimeric form, encodes the toxic domain (Poulsen *et al*, 1991). We used a eucaryotic expression vector that includes the MMTV promoter to obtain a construction with the *gef* gene. Transfection of this construct into MCF-7 cells and the induction of these tumoral cells with Dex led to a marked decrease in the rate of cell growth in MCF-7TG cells in comparison with parental MCF-7 cells. This finding suggests that the *gef* gene has an antiproliferative effect on breast cancer cells.

Although the *gef* gene showed considerable cytotoxic effects in MCF-7 cells, it is not clear how this gene inhibits growth of these tumour cells. Nonmammalian genes may act through different mechanisms when they are introduced and expressed in tumour cells. However, the induction of apoptosis in tumour cells is a frequent mechanism of cell damage. Gene therapy strategies based on the use of diphtheria toxin A gene (Paulus *et al*, 1997) have achieved complete inhibition of protein synthesis in glioblastoma cells. The chicken anaemia virus-derived protein VP3 (apoptin) is able to induce programmed cell death in transformed and malignant cells, including osteosarcoma cells (Zhuang *et al*, 1995; Pietersen *et al*, 1999). In melanoma, tranfection of the transcription factor E2F-1 induced apoptosis in the tumoral cell lines SK-MEL-28 and SK-MEL-2 (Dong *et al*, 1999). To determine whether apoptosis is also the mechanism by which the *gef* gene induces cytotoxicity, MCF-7TG cells were stained with annexin V and PI, and examined with fluorescence-activated cell sorting. These studies clearly demonstrated that cytotoxicity was the result of induction of apoptotic cell death. However, apoptosis was clearly demonstrated after 48 h of induction, in contrast to what has been observed in procaryotic cells such as *Pseudomonas putida* (Ronchel *et al*, 1998). In procaryotic cells, widespread cell death occurred 7 h after induction. This may reflect structural and functional differences between human and bacterial cell membranes or to the need for a target amount of Gef protein in the eucaryotic cells. This latter requirement has been observed in procaryotic cells, in which a critical concentration of Gef protein (different from other killer proteins such as protein E) was needed to provoke cell death (Ronchel *et al*, 1998). In MCF-7TG, apoptosis was confirmed in electron microscopic images. The most significant finding was the compaction and marginalisation of chromatin at the nuclear periphery, which is a cytological hallmark of apoptosis (Kerr *et al*, 1995). Similar changes have been observed during apoptotic cell death in MCF-7 cells transfected with antisense Hsp70 cDNA (Nylandsted *et al*, 2000).

In procaryotic cells, it has been shown that the product of the *gef* gene is anchored to the cell membrane, where it diminishes the membrane potential and leads to membrane leakiness, which in turn results in the efflux of cellular Mg^2+^ and the influx of periplasmic molecules (Gerdes *et al*, 1986). This mechanism induces morphological changes in cells, which subsequently undergo cell death (Ono *et al*, 1987). The specific mechanisms of action of the *gef* gene in eucaryotic cells have not been elucidated. Nonetheless, two of our findings stand out. Firstly, cell cycle analysis in MCF-7TG cells suggested that *gef* gene expression is associated with aberrant cell cycle control characterised by premature progression from G_1_ to S phase and G_2_ delay. The results of cell cycle analysis are consistent with those in other tumour cell lines in which apoptotic mechanisms have been induced (Wei *et al*, 1998). Since they are unable to accomplish normal mitosis, these cells do not proliferate, and apoptotic cell death is induced. Secondly, we also observed a typical pattern of mitochondrial dilation that was always present in all MCF-7TG cells analysed after *gef* expression was induced. This finding may be related to the induction of apoptosis by the *gef* gene, and may also be related to the alteration in mitochondrial membranes, which has been demonstrated to be a critical step in several apoptotic pathways (Boya *et al*, 2001). However, although numerous models of apoptosis have been described in tumour cells (e.g., calcium-binding proteins, protein kinases, nuclear transcription factors, oncogenes and cysteine proteases) (Rowan and Fisher, 1997; Favrot *et al*, 1998), the pathway by which the *gef* gene acts in MCF-7 cells presently remains under investigation.

To our knowledge, this is the first demonstration that the *gef* gene, which has a killing function in *E. coli*, significantly impairs proliferation in tumoral MCF-7 cells derived from breast cancer. Although the mechanism remains unknown at present, it is clear that the product of the *gef* gene induces cellular modifications that are representative of apoptosis in these tumour cells. Our results suggest that the *gef* gene can be considered a possible candidate for a new gene therapy strategy, which is simpler than other killer-suicide systems because it does not require the use of a prodrug. The possibility of using selective transcriptional control sequences with *gef* therefore offers the gene therapist a tool of significant potential. We are carrying out experiments in mice and in other tumoral cells which can determine the effectiveness of this new cancer therapy approach.
